# Nanostructured biocomposite films of high toughness based on native chitin nanofibers and chitosan

**DOI:** 10.3389/fchem.2014.00099

**Published:** 2014-11-18

**Authors:** Ngesa E. Mushi, Simon Utsel, Lars A. Berglund

**Affiliations:** ^1^Department of Fiber and Polymer Technology, Royal Institute of TechnologyStockholm, Sweden; ^2^Department of Fiber and Polymer Technology, Wallenberg Wood Science Center, Royal Institute of TechnologyStockholm, Sweden

**Keywords:** chitin nanofibers, chitosan, nanostructured, nanocomposites, mechanical properties

## Abstract

Chitosan is widely used in films for packaging applications. Chitosan reinforcement by stiff particles or fibers is usually obtained at the expense of lowered ductility and toughness. Here, chitosan film reinforcement by a new type of native chitin nanofibers is reported. Films are prepared by casting from colloidal suspensions of chitin in dissolved chitosan. The nanocomposite films are chitin nanofiber networks in chitosan matrix. Characterization is carried out by dynamic light scattering, quartz crystal microbalance, field emission scanning electron microscopy, tensile tests and dynamic mechanical analysis. The polymer matrix nanocomposites were produced in volume fractions of 8, 22, and 56% chitin nanofibers. Favorable chitin-chitosan synergy for colloidal dispersion is demonstrated. Also, lowered moisture sorption is observed for the composites, probably due to the favorable chitin-chitosan interface. The highest toughness (area under stress-strain curve) was observed at 8 vol% chitin content. The toughening mechanisms and the need for well-dispersed chitin nanofibers is discussed. Finally, desired structural characteristics of ductile chitin biocomposites are discussed.

## Introduction

**Figure 1:**
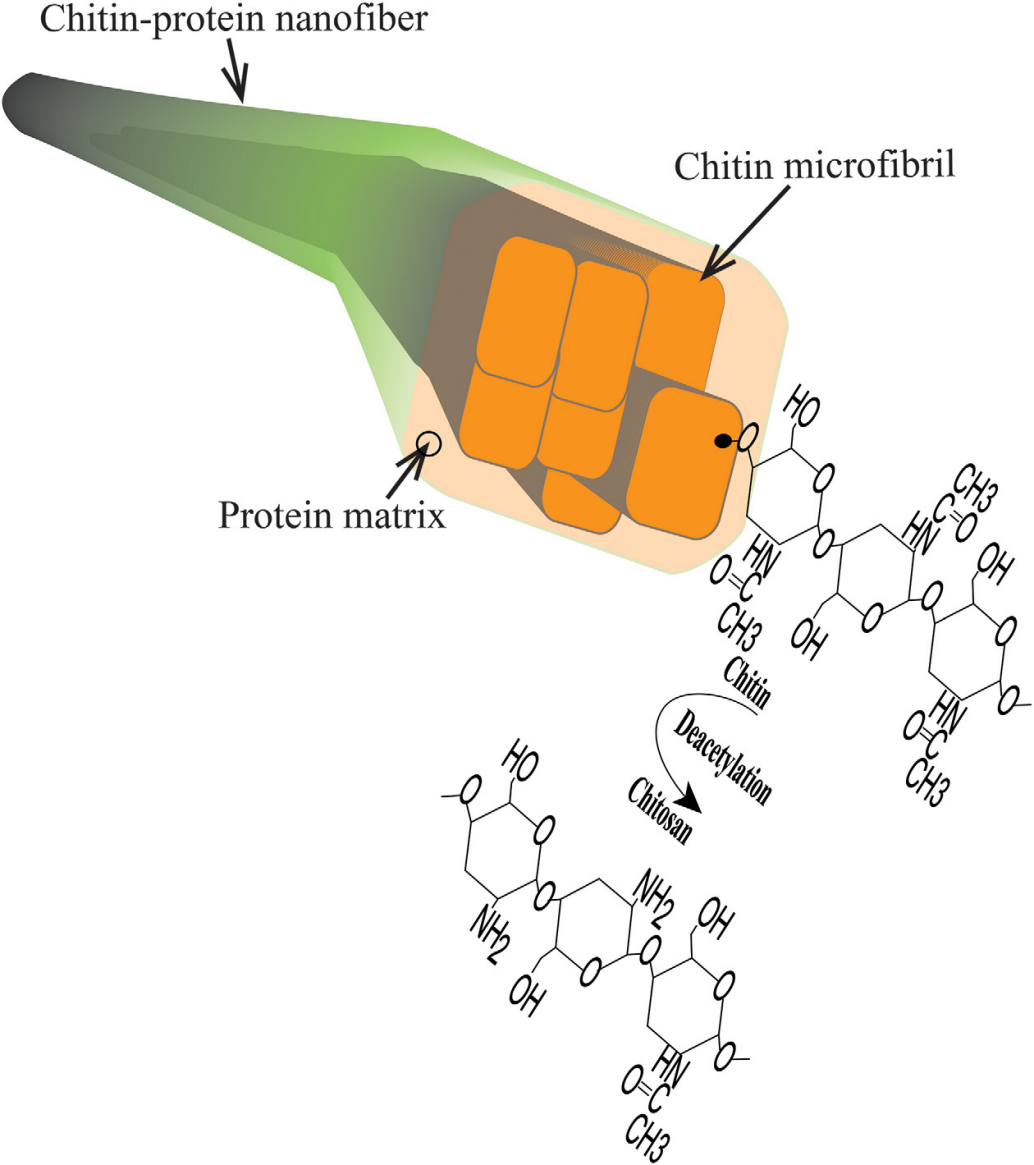
**Schematic diagram of chitin structures including chitin-protein nanofiber, chitin microfibril, chitin polymer chain and chitosan**.

Chitosan is a widely used biopolymer and interesting for use in packaging and biomedical applications. It is commercially available as a derivative of chitin microfibrils from crustaceans. The chitin molecule itself consists of N-acetyl glucosamine units. The preparation of chitosan then involves derivatization through elimination of the chitin acetyl group, and the final sugar monomer is N-glucosamine. In biological organisms, chitin is predominantly organized in extended chain conformation and assembled in the form of microfibrils (Figure 1). This structural organization is vital for the mechanical function of cuticles and exoskeletons of insects and crustaceans (Neville, 1967; Raabe et al., 2005). In addition, chitin structures provide support for tissues and organs such as muscles, eyes, throat etc. Chitosan is in nature less common, but is present as a cell wall component of filamentous fungi, where chitosan biosynthesis is through deacetylation of chitin (Bartnicki-Garcia, 1968; Muzzarelli et al., 2012).

Recently, chitin nanocrystals have been considered for nanocomposites (Gopalan Nair and Dufresne, 2003; Sriupayo et al., 2005; Mathew et al., 2009). Chitin nanowhiskers were combined with chitosan (Sriupayo et al., 2005; Shelma et al., 2008), polycaprolactone (Ji et al., 2012) or poly (vinyl alcohol) (Lee et al., 1996) to improve the mechanical properties of the polymer. One reason for the interest in chitin and chitosan is favorable wound healing properties (Yusof et al., 2003; Shelma et al., 2008; Murakami et al., 2010). However, mechanical properties of neat chitosan films leave room for improvement, as can be concluded from data in published studies; Young's modulus *E* = 2.4 GPa (Ifuku et al., 2013), tensile strength σ^*^ = 40–100 MPa (Mima et al., 1983; Park et al., 2002; Ifuku et al., 2013) and strain to failure ε^*^ = 6–100% (Mima et al., 1983; Park et al., 2002; Shelma et al., 2008; Fernandes et al., 2010). One may note the wide range in strain to failure due to differences in molar mass, environmental conditions and casting conditions. The mechanical properties of nanocomposites based on chitin nanowhiskers combined with polymer matrices such as chitosan (Sriupayo et al., 2005; Shelma et al., 2008), poly methylmethacrylate (Chen et al., 2014), poly (vinyl alcohol) (Lee et al., 1996) and polycarprolactone (Ji et al., 2012) have also been reported. The mechanical properties of these nanocomposites are generally low; strength σ^*^ = 84 MPa, strain to failure ε^*^ = 9% at 3% whisker content (Sriupayo et al., 2005); modulus *E* = 1.6 GPa, σ^*^ = 60 MPa and ε^*^ = 7% at 17% whisker content (Shelma et al., 2008). Chemical cross-linking was also used to improve some mechanical properties of the nanocomposites; for example, chitin nanowhisker-chitosan scaffolds cross-linked via amine groups (Mathew et al., 2009). It is not clear why chitin nanowhisker reinforcement effects are so small, although there are several possible explanations. The aspect ratio is small with lengths in the range of 200–500 nm and diameters 6–20 nm (Yamamoto et al., 2010), the chitin content is often low and agglomeration effects may be present.

An interesting recent development is the extraction of chitin from crustaceans in the form of long nanofibers (Ifuku et al., 2010; Mushi et al., 2014a). The chemical and physical properties are very attractive; degree of acetylation = 87%, diameter = 3–6 nm, length = 800–1000 nm. The aspect ratio (length/diameter) is in the same range as for cellulose nanofibers disintegrated from wood pulp (>100) (Henriksson et al., 2007). In preparation of chitin nanowhiskers (nanocrystals), treatment with concentrated HCl or NaOH leads to formation of shorter rods. In the present chitin nanofiber structure, the fibrous structure is much longer and possibly contain disordered regions. From a basic science point of view, it is interesting to compare with suspensions and polymer matrix nanocomposites based on fibrous nanocellulose. Effects from different structure and surface characteristics of fibrils as well as intrinsic fibril strength may be possible to estimate. In a more practical sense, chitin nanofibers can be used as a reinforcement phase in chitosan in order to improve the mechanical properties. Compared with chitin nanowhiskers, it may be possible to use higher chitin content and to better control the degree of fibril dispersion.

There are two major purposes of reinforcing chitosan-based films with nanofibers. First, to improve mechanical properties such as strength, modulus and toughness. For food packaging films, tensile strength above 50–70 MPa and high toughness are desirable (Chambi and Grosso, 2011). A second purpose is to reduce effects from the moisture affinity of chitosan. This include moisture sorption, swelling and reduced barrier properties. In the present study, focus is on mechanical properties. The first attempt to reinforce chitosan with high aspect ratio bio-nanofibers was in a well-cited study by Fernandes et al. (2010) where cellulose nanofibers were used. The nanocomposite films showed high optical transparency, a Young's modulus of about 6.8 GPa and a strength of 115 MPa at 60% volume fraction of cellulose (Fernandes et al., 2010). However, ductility was sacrificed. More recently, Ifuku et al. (2013) reported high strength (e.g., σ^*^ = 140 MPa at 80 wt.% nanofiber content) for chitin-chitosan nanocomposites (Ifuku et al., 2013). The study employed a deacetylated chitin nanowhiskers/nanofibers concept. Chitin nanofibers with a deacetylated surface were combined with a chitosan matrix. The focus of the present study is to discuss strain to failure (in particular toughness) and deformation mechanisms. The ductility (strain to failure) of composites was observed to be lower as compared to neat chitosan-based films (Ifuku et al., 2013). The nanocomposite films based on deacetylated chitin nanofibers and chitosan showed slightly better modulus and strength (*E* = 7.8 GPa and σ^*^ = 125 MPa at 60 wt.% nanofiber content, Ifuku et al., 2013) as compared to results from nanofibrillated cellulose and chitosan (*E* = 6.8 GPa and σ^*^ = 115 MPa at 60 vol% nanofiber content) (Fernandes et al., 2010).

In the present study, the possibilities to combine strength and ductility in order to obtain high work to fracture (area under stress strain curve) are in focus. The chitin nanofibers are different from those reported in earlier studies (Ifuku et al., 2013). The present origin is lobster rather than crab, and the present nanofibers have lower protein content and higher degree of acetylation, see Mushi et al. (2014a). The present study discusses the importance of the colloidal state and suggests routes toward material compositions and nanostructures with even higher toughness, based on observed deformation behavior. We emphasize the importance of colloidal stability and report low composite moisture sorption due to the favorable chitin/chitosan interface. The resulting nanocomposites show considerable ductility and toughness. This is related to the intrinsic chitosan ductility and the well-dispersed nanostructured network of chitin nanofibers in the final material. The chemical chitin-chitosan compatibility is also an important factor. The use of chitosan allows for compositional tailoring (chitin content and degree of acetylation) to meet requirements in a variety of packaging or wound healing applications.

## Materials and methods

### Materials

Low protein native chitin nanofibers were disintegrated from lobster *Homarus Americanus* of Northwest Atlantic, produced in Canada, according to the procedure reported in our previous work (Mushi et al., 2014a). The lobster was cleaned to take away salts and tissues. Demineralization to remove calcium carbonate minerals was performed with 2 M HCl twice for a duration of 1 h in each step. In the first step, treatment was done on large exoskeleton pieces to reduce dust from mineral particles during grinding. The sample was freeze dried. It was crushed with a 500 μm mesh size (Retsch grinder, Model ZM200, Germany) to produce crude chitin powder. Second, demineralization was performed on the freeze dried crude chitin powder. Depigmentation was followed by washing for 12 h overnight with ethanol (96%). Lastly, protein was removed by treatment with 20% NaOH for 2 weeks. The chitin sample was washed in deionized water between each step. Another washing step was performed with 4% acetic acid until the suspension of chitin powder turned whitish. The white creamy suspension of chitin powder was mechanically treated in a blender (VM0105E, USA). It was homogenized through a Microfluidizer (M-110EH, Microfluidics Ind., Newton, MA, USA) so that a translucent hydrocolloid of chitin nanofibers was obtained. Degree of acetylation, DA, ranged between 86 and 87% based solid state ^13^C NMR (Nuclear Magnetic Resonance Spectroscope). Chitosan powder from shrimp (high viscous, Sigma, Germany) with a degree of acetylation of less than 15% was used. It was dissolved in acetic acid (1.0 wt.%), and aggregates where removed by centrifugation (4000 rpm, 10 min, room temperature).

### Dynamic light scattering (DLS)

The zeta potential (ζ) and aggregate size of the chitin nanofiber hydrocolloid was studied by dynamic light scattering using Zetasizer Nano, Model ZEN3600 (Malvern Instruments Ltd., UK). The light source was operated at a wavelength of 633 nm. The chitin nanofiber suspension was diluted to a concentration of 50 mg/L at pH 3 and filled in a PMMA (Poly Methyl Methacrylate) cuvette and scanned three times at ambient conditions (i.e., 25°C).

### Quartz crystal microbalance (QCM)

A Quartz Crystal Microbalance Model QCM-E4 from Q-Sense AB (Västra Frölunda, Sweden) was used to study chitosan adsorption to a chitin nanofiber surface with a continuous flow of 100 μL/min (Marx, 2003). The crystals were AT-cut quartz crystals with a 5 MHz resonance frequency and an active surface of sputtered silica. These were rinsed with Milli-Q water, ethanol and Milli-Q water, dried in nitrogen, and then placed in an air plasma cleaner (Model PDC 002, Harrick Scientific Corporation, NY, USA) under reduced air pressure for 120 s and 30 W. A 1 g/L chitin nanofiber suspension was spin-coated on the cleaned crystals resulting in a fully covered chitin nanofiber surface. The change in frequency can be used to estimate the change in adsorbed mass according to the Sauerbrey model Equation (1) (Sauerbrey, 1959).

(1) m=C × Δf/n

where, *m* is the adsorbed mass per unit area (mg/m^2^), *C*, the sensitivity constant = −0.177 [mg/(m^2^ · Hz)], Δ*f*, the change in resonant frequency (Hz), and *n* is the overtone number.

### Preparation of nanostructured composites

A colloidal suspension of ca. 1 wt.% solid content of chitin nanofibers and a chitosan solution in at least 4% acetic acid (initially the concentration of acetic acid was 1 wt.%) was slowly mixed under magnetic stirring for 12 h overnight to allow a uniform mixture. Casting was done on a Teflon film surface securely clamped to a glass cylinder with a diameter of 72 cm. This technique was employed for the preparation of nanopaper membranes in previous studies (Henriksson et al., 2008; Sehaqui et al., 2010; Mushi et al., 2014b). Pure chitin films were sensitive to moisture, so controlled drying of the composite film was performed in the presence of excess acetic acid and low temperature condition in an oven at 37°C in order to avoid warpage or uneven distribution of chitosan and nanofibers in the solid film. Evaporation of water and acetic acid resulted into a consolidated nanostructured composite film. Several films of the same volume fraction were prepared, and at least two were used for each composition in this study. The previously established nanopaper preparation procedure was employed for the preparation of nanopaper membranes (Sehaqui et al., 2010; Mushi et al., 2014b).

### Structural characterization

Structural characterization of the nanostructured composite was performed in a Field Emission Scanning Electron Microscope (FE-SEM) S-4800 (Hitachi). The sample was conditioned in a desiccator for 12 h overnight to remove moisture and then platinum-palladium sputtered in Agar HR Sputter Coater prior to structural imaging in the SEM. Surfaces of the nanostructured chitin membrane and composite were studied and a secondary electron detector was employed for capturing images at 1 kV. Porosity determination was based on the density method reported in previous work (Mushi et al., 2014b). Void content, *V*_*v*_, was deduced from Equation 2. Equation 3 is the theoretical density, ρ_*c*_, of void-free composite used in earlier work (Sehaqui et al., 2011). Weight fraction, *W*, was related to volume fraction, *V*, based on Equation 4. The subscripts stand for; *v* - voids, *c* - void-free composite, *f* - chitin nanofiber, *sample* refers to the real composite with voids and *chitosan* is the real matrix without voids. The measured density of chitosan film was considered as a true density of chitosan, ρ_*chitosan*_. The density of dry chitin, ρ_*f*_, is 1.425 g/cm^3^ according to literature (Carlström, 1957).

(2) $$V_{v}=1-\frac{\rho_{sample}}{\rho_{c}}$$

(3) $$\rho_{c}=\frac{1}{\frac{W_{f}}{\rho_{f}}+\frac{\left(1-W_{f}\right)}{\rho_{chitosan}}}$$

(4) $$V_{f}=\frac{W_{f}}{\rho_{f}}\times \rho_{sample}$$

### Mechanical characterization

Tensile tests were performed using Instron Universal Tensile Testing Machine Model 5944 (UK) equipped with a 500 N load cell. The specimens were conditioned in a room with 50% relative humidity and 23°C for 12 h overnight. For each volume fraction, at least five specimens were prepared with width and length of 5–40 mm, respectively. Sample thicknesses were typically 60–80 μm. Tensile tests were performed at a strain rate of 4 mm per min. Mechanical properties such as tensile modulus, E, tensile strength, σ^*^, tensile strain to failure, ε and work to fracture, U were estimated based on conventional analysis of nominal stress-strain curves. Tensile samples were conditioned at 50 and 90% relative humidy (RH) and weighed to analyze the effect of moisture absorption in relation to mechanical behavior. Relative humidity was controlled in a dessicator with various salts and weight change was calculated from ratio of weight before and after saturation in high relative humidity, according to description in previous work (Mushi et al., 2014b). Dynamic mechanical analysis (DMA) was performed in TA Instruments equipment (Model Q800). In this equipment, dynamic heating ranged from −100 to 300°C at a rate of 3°C/min and a frequency of 1 Hz, change in storage modulus and tan δ was recorded. Samples (width = 5 mm, length = 10 mm) were conditioned at 80°C for 10 min in order to stabilize moisture content.

## Results and discussion

### Chitin nanofiber colloid in chitosan solution

Disintegration of the lobster exoskeleton was successfully performed according to procedure in the experimental section. A viscous transluscent hydrocolloid was obtained at pH 3 in the presence of acetic acid. The structure and composition of the chitin nanofibers were previously reported (Mushi et al., 2014a). Briefly, the average protein content was 4.7, with 95.3% chitin (Mushi et al., 2014a). In the present study, the nanofibers can be described as semiflexible fibrils with an average diameter of 10 nm and an average length of 1 μm. Note that in the biology community, the smallest fibrils are often termed microfibrils. Chitin microfibrils are 3–4 nm in diameter and embedded in proteins to form larger diameter, fibrous chitin aggregates (Raabe et al., 2006; Mushi et al., 2014b). “The present” nanofibers illustrated in Figure 1 are aggregates of several microfibrils and much reduced protein content compared with the native structure. It is likely that the surface of the nanofibers are chitin-rich with some deacetylation, see Figure 1. The chitosan polymer was obtained from Sigma Aldrich, and is a chitin derivative prepared by dissolution and deacetylation of chitin from shrimp exoskeletons. The chitin nanofiber colloid was mixed with the chitosan solution, See Materials and Methods Section.

**Figure 2:**
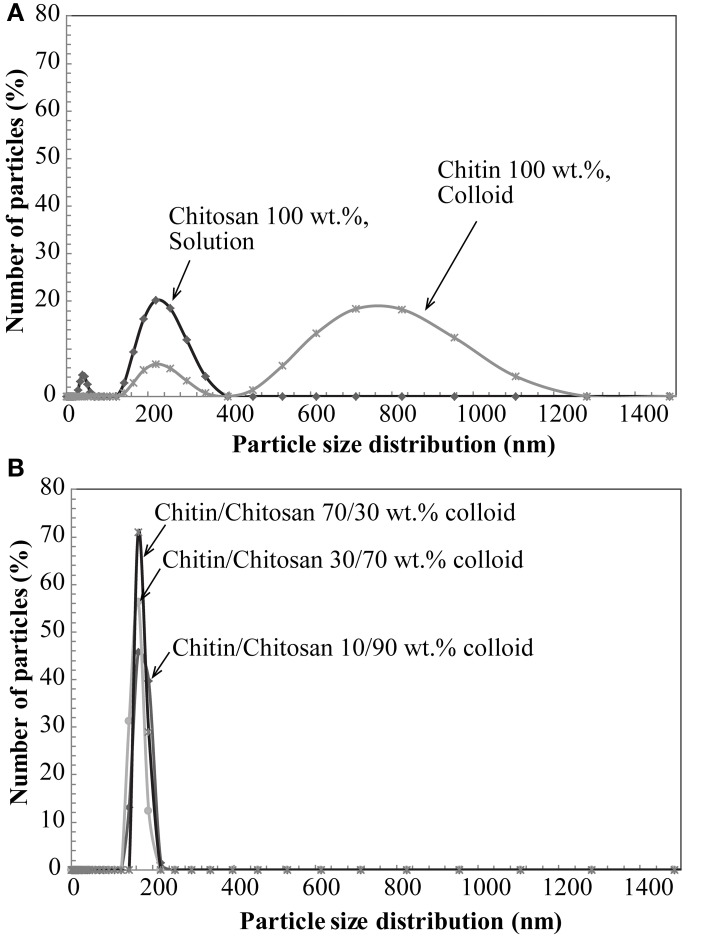
**Nominal aggregate size distribution by DLS; (A) Chitin and chitosan, (B) colloidal suspension based on chitin nanofibers in aqueous chitosan solution**.

The zeta potential and aggregate size of the colloidal chitin-chitosan mixture were estimated for different composites based on DLS data. Zeta potential is an electrokinetic potential between the interfacial double layer of the chitin nanofiber and a reference point in the bulk liquid. Particle size estimations are also based on diffusion rate (Fall, 2013). Table 1 presents the zeta potential and chitin aggregate size data for the chitin nanofiber suspension at different concentrations of chitosan. In Figure 2, the size distribution estimates based on DLS are presented, (**A**) pure chitin colloid and pure chitosan solution and in (**B**) the chitin/chitosan colloidal mixtures. Note that the “size” can only be interpreted as a relative measure at this stage. The zeta potential data are in agreement with previous results by Fan et al. (2012). As an estimate, the threshold value for a stable colloid is ≥+30 or ≤−30 mV. If we compare Figure 2A and Figure 2B, the most apparent effect is that the chitin aggregate “size” decreased from 634 to 165 nm after mechanical mixing with the chitosan solution. The charged chitosan molecules are able to reduce the size of chitin aggregates. The peak at lowest particle size for chitin colloids is believed to originate from the wide distribution in chitin fibril size. Note also that the chitosan solution shows large particles, Figure 2A, which indicate the presence of chitosan agglomerates rather than an ideal solution. In the chitin/chitosan mixtures, Figure 2B, those chitosan agglomerates are no longer present. Also, the small size peak for neat chitin is not present. The DLS data confirm the visible impression that the present chitin-chitosan colloid mixture forms a stable colloidal suspension. This is important since a prerequisite for well-dispersed chitin nanofibers in the solid nanocomposite material is well-dispersed chitin also in the colloid. The data can be compared with zeta potential data for stable TEMPO-oxidized cellulose nanofiber hydrocolloids (from −39 to −52 mV) (Fall et al., 2011; Fall, 2013). The reason for the positive charge on the chitin nanofibers is partial deacetylation, which results in partially chitosan-like nanofiber surfaces.

**Table 1:** **Zeta potential (ζ) and nominal chitin aggregate size (nm) of chitin nanofiber hydrocolloids in chitosan solution with composition expressed as weight fraction**.

Sample description (Chitin wt.%)	100	70	30	10	0
Zeta potential (mV)	+45	+65	+64	+58	+45
Average particle size (nm)	670	295	190	220	220

*Note that particle size is a relative estimate for comparative purposes, since the nanofibers have non-ideal geometry. The dry content was 0.005 wt%. The hydrocolloid contains about 4% acetic acid, and measurements were done at pH 3*.

**Figure 3:**
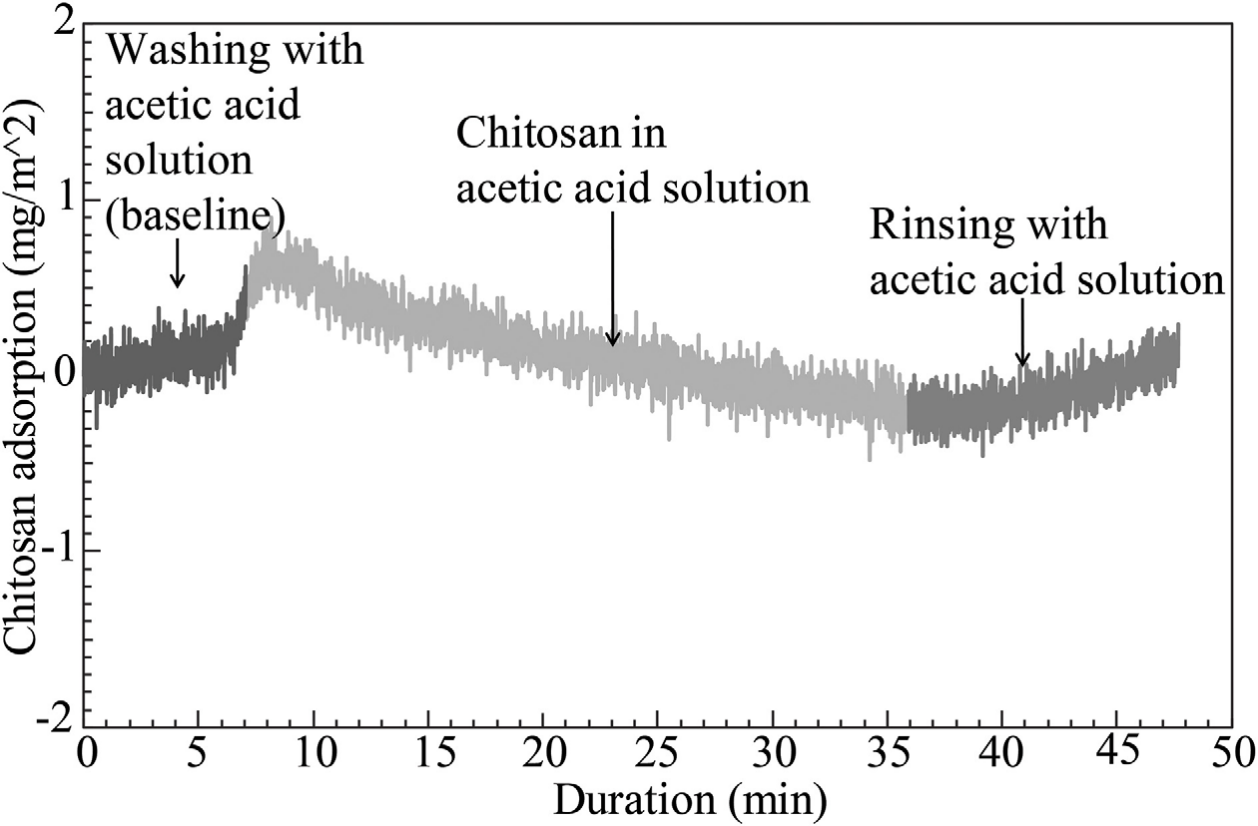
**QCM curve for characterization of chitosan adsorption on spin-coated chitin nanofiber model surface exposed to a chitosan solution at pH 3**.

The combination of chitin nanofibers with chitosan is of specific interest because of the potential for high compatibility (strong molecular interactions) at the nanofiber-polymer matrix interface. The chitin/chitosan charge repulsion in the colloid is apparently positive in that chitin agglomerate size is reduced, see Figure 2. Figure 3 presents QCM results of chitin nanofiber and chitosan mixtures at pH 3. A spin-coated chitin nanofiber model surface was exposed to a chitosan solution (100 mg/L) in acetic acid for 30 min. The baseline was first established with the neat acetic acid solution. The QCM curve in Figure 3 shows no change in baseline with the addition of chitosan during the washing and rinsing steps, and it is concluded that no chitosan is adsorbed. This confirms the charge repulsion phenomenon in the colloid between the chitosan and the chitin nanofiber surface at pH 3.

Due to deacetylation, there is a large concentration of amine groups in the present chitosan (above 85% of the maximum possible content). Although bulk degree of acetylation (DA) in our native chitin nanofibers was between 86 and 87%, the nanofiber surface is much more deacetylated compared with the core. A degree of deacetylation of about 50% was estimated in a previous study (Das et al., 2012). The QCM results in Figure 3 thus correlate well with DLS data and the stable behavior of the chitin-chitosan colloidal mixture. The chitosan did not adsorb to chitin nanofibers due to electrostatic repulsion.

### Preparation of chitin-chitosan nanocomposites

The nanostructured composite film was prepared by a simple film casting procedure where the liquid phase was evaporated. Table 2 presents densities and porosities of the composite films, as well as neat chitosan film and chitin membrane. With the exception of the non-porous chitosan films, porosities are estimated to be in the 13–20% range. In the context of physical properties, the volume fraction of reinforcement is the physically correct parameter, and Table 2 shows a significant difference between weight fraction and volume fractions due to the lower density of chitosan compared with chitin. The highest volume fraction of chitin is 56% with the present preparation procedure and this provides potential for strong property enhancements.

**Table 2:** **Data for density and porosity: Neat chitosan (0 wt.% chitin), chitin/chitosan composites, and neat chitin porous membrane (100 wt.% chitin)**.

Chitin nanofiber weight fraction (%)	0	10	30	70	100
Chitin nanofiber volume fraction (%)	0	8	22	56	84
Density of sample with voids (g/cm^3^)	1.22	1.08	1.03	1.13	1.21
Density of void-free composite (g/cm^3^)	1.22	1.24	1.28	1.36	1.425
Porosity (%)	0	13	20	17	16

The purpose was to study ductility of an all-chitin-based composite based on chitin nanofibers in a chitosan matrix. The liquid phase is the water-acetic acid mixture. Slow evaporation was carried out in order to reduce warpage from concentration gradients of water-acetic acid. The state of swelling in a local region depends on water-acetic acid concentration, so that large through-thickness differences in concentration and swelling strains can cause warpage. The effect of acetic acid on chitosan may show similarities to the effect of glycerol on starch films. The presence of acetic acid was reported to induce conformational changes in chitosan conformations (Kienzle-Sterzer et al., 1982), so that the ductility was improved.

### Structural characterization

**Figure 4:**
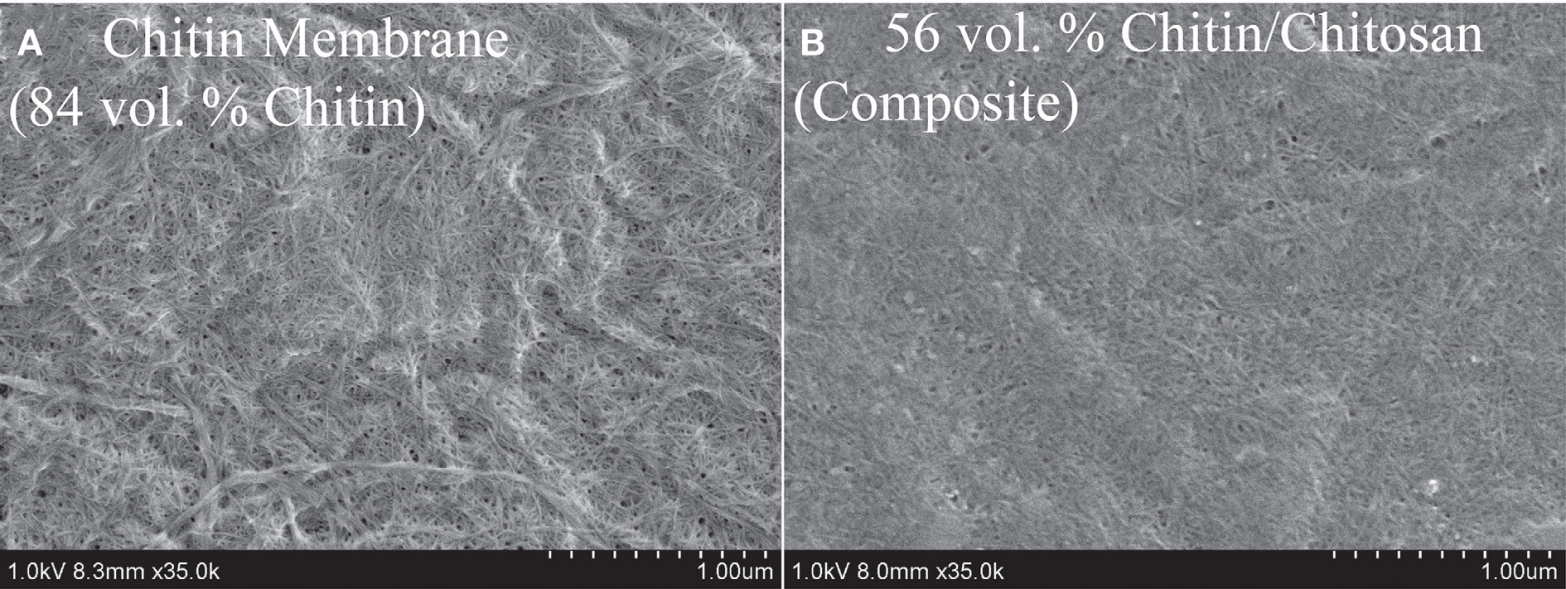
**SEM topographical view of (A) 84 vol.% porous neat chitin membrane, and (B) 56 vol.% chitin/chitosan nanocomposite (also porous)**.

The FE-SEM micrograph in Figure 4A presents the upper surface of a porous neat chitin nanofiber membrane. The nanofiber population contains both small nanofibers with diameters at a scale of ten nm, as well as larger agglomerated nanofiber bundles with diameters at the 100 nm scale. The nanofibers have curved geometries primarily random in-plane and to some extent out-of-plane. Pores at a typical scale of 10–40 nm are apparent as dark regions and there is considerable surface roughness. Figure 4B is the surface of the nanostructured chitin-chitosan matrix composite. The chitin nanofiber network is still apparent at a chitin volume fraction of 56%. According to data in Table 2, the bulk porosities are comparable (17% in **B** and 16% in **A**) and pores are visually apparent in Figure 4. The chitosan matrix in Figure 4B appears to be well distributed.

### Uniaxial tensile properties

**Figure 5:**
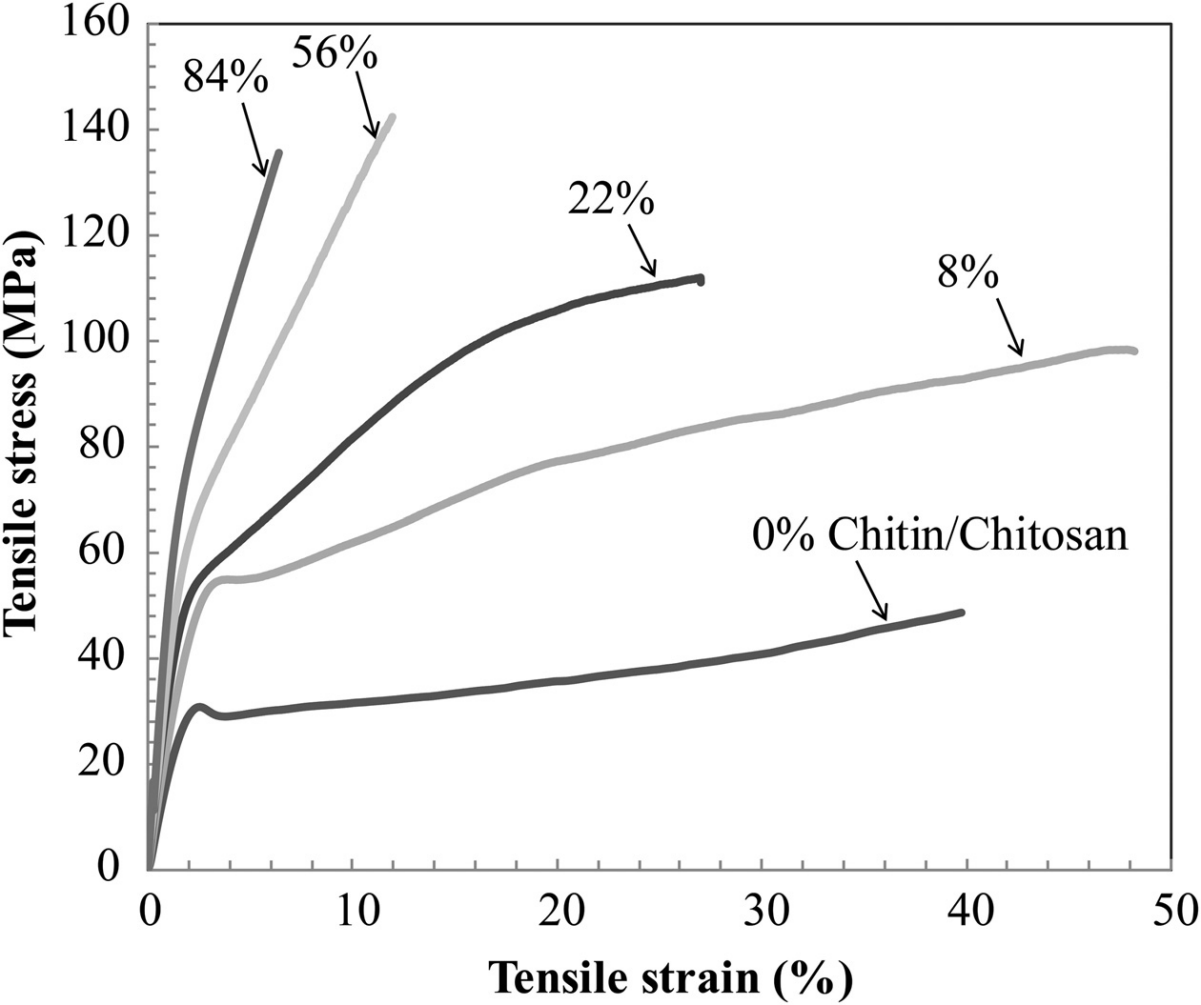
**Uniaxial tensile stress-strain curves of nanostructured composites and reference materials**. *V*_*f*_ stands for volume fraction of chitin nanofibers.

Figure 5 presents the stress-strain curve of the nanostructured chitin membrane (*V*_*f*_ = 84%) and the nanostructured composites (*V*_*f*_ = 8, 22, 56%) as well as data for neat chitosan films. The most important observation is that the nanostructured composite (*V*_*f*_ = 8, 22, 56%) shows high strain-to-failure for all compositions. Strain-hardening is observed in the post-yield region for 84, 56, 22, and 8%. For the other materials, there is initial strain-softening, followed by strain-hardening associated with chitin nanofiber network reorientation. The behavior is analogous to cellulose nanofiber composites (Sehaqui et al., 2011). However, at 8 and 22% chitin volume fraction, two plastic deformation regions are apparent. In a previous study of cellulose nanofibers in hydroxyethyl cellulose matrix, the second plateau was assigned to plastic deformation in matrix-rich regions between nanofiber-rich lamellae (Sehaqui et al., 2011). The 8% composition is interesting. The yield strength (stress at onset of non-linear behavior) increases strongly compared with the neat chitosan (from 32 to 51 MPa, see Table 3). Most likely, composite yielding is associated with onset of chitosan shear yielding. The global yield stress is strongly increased for composites due to the load-carrying capability (stiffness) of the chitin nanofiber network (local chitosan stress becomes much lower than the global composite stress). In addition, the strain-to-failure is even higher than for neat chitosan. One may speculate that failure is associated with growth of nanoscale voids, and this process is delayed to higher strains due to the presence of the chitin nanofiber network. For the *V*_*f*_ = 22% composition, strain to failure is decreased compared with *V*_*f*_ = 8%. One may note that for *V*_*f*_ = 22%, the stress level is much higher at a given strain in the plastic region, and this is likely to cause failure at lower strain. Some of the chitin nanofibers are subjected to very high local stress, which is much higher than the average nanocomposite global stress. This will result in local chitin nanofiber fracture and lowered strain to failure. For the nanostructured neat chitin membrane, the stress-strain curve shows yielding associated with inter-nanofiber separation dominated by opening tension or shear stresses at the local scale. Then follows substantial strain-hardening associated with nanofiber reorientation and interfibril slippage. This behavior has been discussed in previous studies on cellulose and chitin nanofiber membranes (Svagan et al., 2007; Henriksson et al., 2008; Sehaqui et al., 2011; Mushi et al., 2014a). Table 3 summarizes the mechanical properties of the present materials. The observed nanocomposite ductility is very large, and due to the strain-hardening behavior, the work to fracture (defined as the as the area under the stress-strain curve) also becomes very high. It simply means that substantial mechanical energy is required to cause final fracture. The highest work to fracture values are obtained for the 8 and 22 vol.% chitin compositions.

**Table 3:** **Tensile properties of nanostructured composites and reference materials (nanostructured neat chitin membrane and neat chitosan film; 84% means neat chitin membrane with 16% porosity, 0% means neat chitosan)**.

Chitin content (vol.%)	0	8	22	56	84
Young's modulus (GPa)	2.2 (0.2)	3.2 (0.6)	4.3 (0.2)	5.4 (0.7)	7.3 (0.4)
Tensile strength (MPa)	52 (5)	98 (3)	114 (3)	141 (3)	153 (11)
Yield strength (MPa)	32 (1)	51 (3)	53 (2)	63 (4)	70 (2)
Tensile strain to failure (%)	42 (2)	46 (4)	24 (5)	11 (1)	8 (1)
Work to fracture (MJm^−3^)	16 (0.2)	35 (2)	22 (3)	12 (0.2)	8 (0.2)

*The numbers in bracket are standard deviation*.

**Figure 6:**
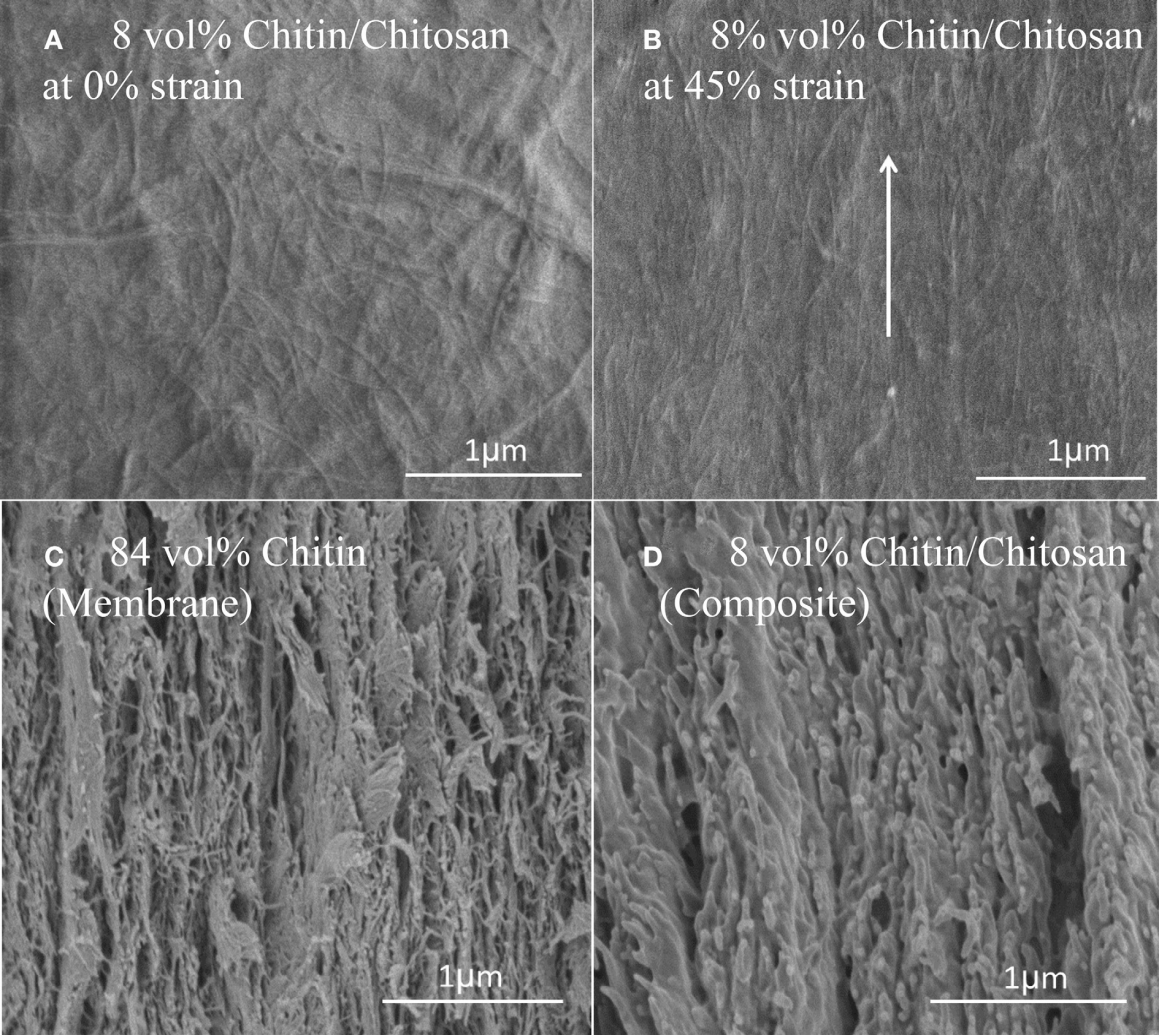
**FE-SEM micrographs of the *V*_*f*_ = 8% chitin composite surface: (A) at 0% strain (B) at 45% strain-to-failure**. Fractured cross section FE-SEM micrographs **(C)** *V*_*f*_ = 84% chitin **(D)** *V*_*f*_ = 8% chitin. The arrow indicates loading direction and major fibril orientation direction after deformation.

Figure 6 shows SEM fracture micrographs of a nanostructured composite (*V*_*f*_ = 8%) and the nanostructured neat chitin membrane. Figure 6A is a topographical image of the nanocomposite film surface at 0% strain. The comparable smoothness of this surface corresponds to the high chitosan content. The estimated small-scale porosity is still substantial (13%). The micrograph in Figure 6B shows the film surface close to the fracture plane at 45% strain after mechanical testing. Substantial chitin nanofiber reorientation is apparent so that the nanofibers are preferably in the direction of uniaxial loading. Figures 6C,D present the cross-sectional fracture surfaces. Chitin fibrils are observed as fine protrusions on the fractured surface. Figure 6C shows the nanostructured chitin membrane and Figure 6D the nanocomposite (*V*_*f*_ = 8%). For the nanostructured membrane, although the structure appears layered, this layering is less distinct than for cellulose nanopaper (Henriksson et al., 2008). The fracture surface is rough, and there are indications of fracture and pull-out of layers from adhering layer neighbors. For the nanocomposite in Figure 6D, the fracture surface is more smooth, and the apparent fracture surface layering indicate that layer fracture is important. Fractured chitin nanofibers with diameters at the scale of tens of nanometers are apparent, although the nanofiber pull-out lengths are very short. Signs of substantial matrix plasticity are apparent in the smooth lamellae surfaces. There are similarities with fracture surfaces in cellulose nanofiber composites with plasticized starch matrix in terms of layered structure, fractured fibers of short pull-out lengths, plastic deformation features of the matrix (Svagan et al., 2007) and reorientation of nanofibers (Sehaqui et al., 2012).

**Figure 7:**
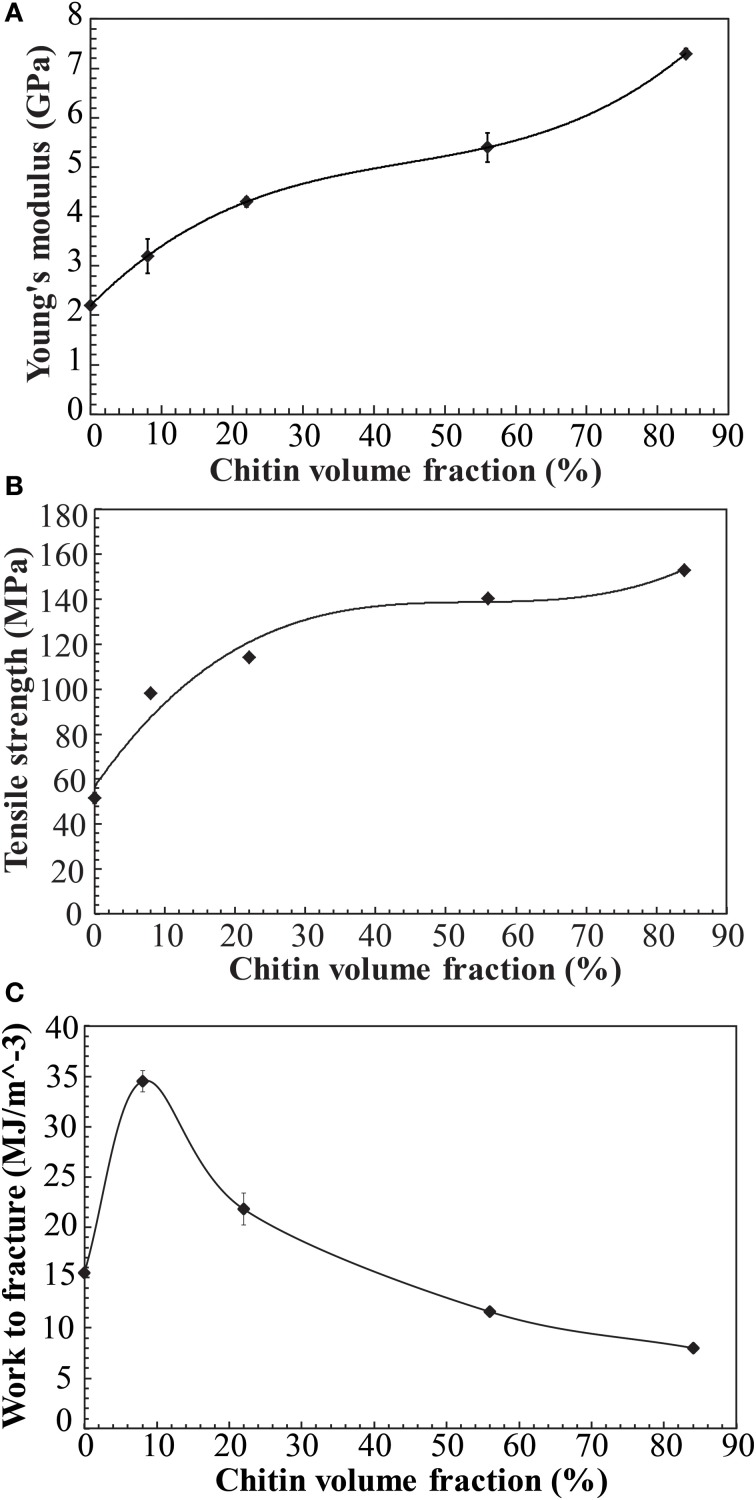
**Mechanical properties vs. chitin volume fraction in the nanostructured composites and reference materials (A) Young's modulus (B) Tensile strength (C) Work to fracture (note that materials have some porosity, see Table 2)**. Solid lines are fit to data.

From Table 3, it was observed that as chitin volume fraction increases, the modulus and strength are increased. In Figures 7A–C, tensile modulus, strength and work to fracture for chitin materials are plotted as a function of chitin volume fraction. There is relatively stronger property increase at lower volume fraction. For a given fiber orientation distribution, tensile modulus depends on intrinsic modulus of constituents and the fiber volume fraction. There is a relatively weaker reinforcement effect at higher volume fractions, possibly due to chitin agglomeration. If chitin is present in the form of localized porous floc network entities, the reinforcement efficiency will be lower than for individually dispersed nanofibers in a polymer matrix. Toughness expressed as “work to fracture”, the area under the stress-strain curve is as high as 35 MJ/m^3^ with 46% strain to failure at a volume fraction of 8% chitin. The use of acetic acid is important, since it can improve solubility of chitosan in water. The strength of chitosan-based films have been reported to depend on acetic acid content and solvent type (Park et al., 1999, 2002) but also degree of acetylation and chitosan molar mass (Mima et al., 1983). Higher solubility leads to more favorable chitosan conformations in the solid composite film and correspondingly higher strength. Higher molar mass also increases strength through increased effects from physical entanglements of chitosan molecules. According to Park et al. (2002), tensile strength and strain to failure of chitosan films increased from 69 to 150 MPa and 4.1–76%, respectively, when 2% acetic acid was added. Again, the most likely reason is improved chitosan solubility and more favorable chitosan-chitosan mixing as well as more favorable chitosan conformations in the film. Chitin nanofiber colloidal properties also depend on molecular interactions (Qi et al., 2013) and this influences the degree of dispersion and the nanostructural details of the film. Poor dispersion in the collolid leads to agglomerate formation which may act as defects in the film so that the strain to failure is decreased.

Moisture sorption data are presented in Table 4. It is interesting to note that the chitin/chitosan nanocomposites show lower moisture content than neat chitosan as well as neat chitin nanofiber membranes. For chitin moisture sorption, the chitin nanofiber surface is the main site for water molecule sorption. The question is then how a chitosan matrix can reduce chitin-related moisture sorption. If hydroxyls and other sites at the chitin microfibril surface are interacting strongly with the chitosan matrix, potential sites for water molecules become occupied. As a consequence, the total moisture sorption of the composite will be lower than rule of mixture predictions, as has been demonstrated for composites based on cellulose nanofibers and epoxy (Ansari et al., 2014). One may thus speculate that chitin-chitosan interfacial interaction at molecular scale decreases the density of sites for moisture sorption. With nanoscale fibrils, the specific surface area is very large and interface effects are therefore very strong. Although hygromechanical or thermomechanical strains may influence sorption (Autran et al., 2002; Wan et al., 2005), such effects have not been considered. One reason is that steady-state conditions are reached fairly rapidly in thin films.

**Table 4:** **Moisture content of the nanostructured composites and reference materials at 50 and 90% RH**.

Sample description (chitin vol.%)	0	8	22	56	84
Moisture content at 50%RH	15.6	8.4	8.2	7.4	10.6
Moisture content at 90%RH	34	16.8	18.1	19.7	22.9

*Note that the 84 vol.% composition is a neat chitin nanofiber membrane*.

The current data confirm that chitin nanofiber composites show much better mechanical properties compared to chitosan reinforced with chitin whiskers (Sriupayo et al., 2005; Shelma et al., 2008), and also somewhat better properties compared to chitosan composites based on cellulose nanofibers (Fernandes et al., 2010). The deformation mechanisms have been clarified. Compared to the previously reported deacetylated chitin nanofiber-chitosan composites (Ifuku et al., 2013), the present data combine similar strength with the added advantage of high ductility and work to fracture. Chitin nanofibers were not strongly deacetylated as in the study by Fan et al. (2012), where the chitin nanofiber surface was deacetylated to chitosan. The toughness data of the chitin/chitosan composites improve our understanding on the importance of chitin dispersion and chitin-chitosan interaction. The chitin-chitosan-acetic acid combination is also interesting. The work to fracture is similar or slightly better than that of nanostructured composites based on cellulose nanofibers (Sehaqui et al., 2011) (maximum work to fracture ≈28 MJ/m^3^). The cellulose nanofibers provide higher strength and modulus, most likely due to better intrinsic strength, stronger interfibril interaction and lower porosity. One may also note that the chitin crystal has lower intrinsic modulus (Ogawa et al., 2011a,b) than cellulose (Sakurada et al., 1964).

### Dynamic mechanical properties

**Figure 8:**
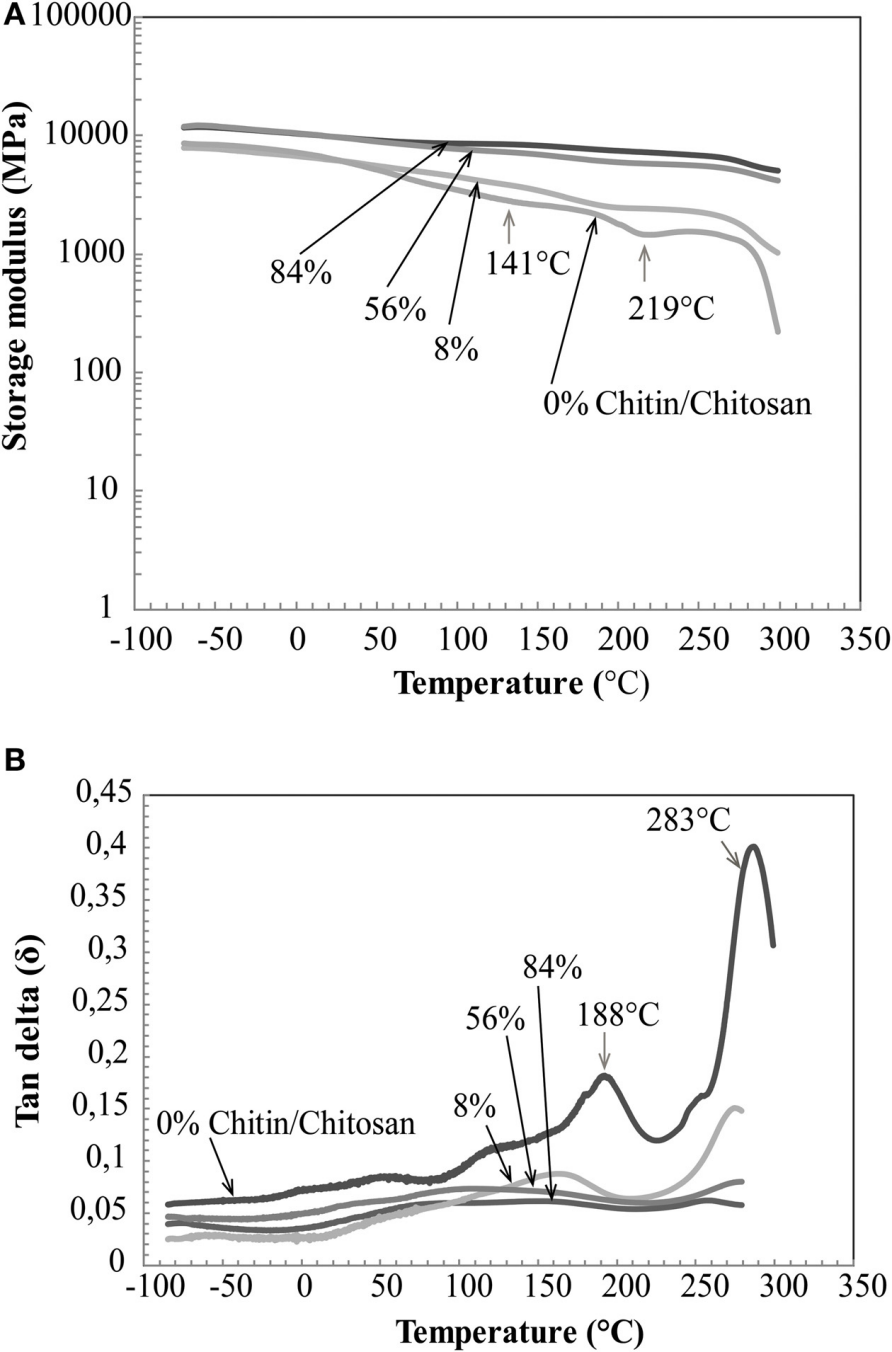
**DMA properties of nanostructured chitin composites (56, 8% chitin by volume), neat chitin membrane (84% chitin by volume) and neat chitosan (0% chitin). (A)** Storage modulus vs. temperature and, **(B)** tan delta vs. temperature.

DMA properties of the nanostructured composites are presented in Figure 8. Previously, Ogura et al studied cast chitin films obtained by dissolution and regeneration(Ogura et al., 1980). It was concluded that chitin degrades thermally prior to its glass transition. In the same study, dry chitosan was reported to show a Tg of around 140°C. In Figure 8A, the thermal stability of chitin network materials is apparent. A gradual decrease of storage modulus with chitin volume fraction and temperature is observed. This is expected, since the chitin nanofiber has much higher modulus than chitosan. The difference in storage modulus between the porous chitin membrane (84% by vol. of chitin) and the chitin/chitosan nanocomposite (56% by volume of chitin) is very small. For chitosan (0% by volume chitin) a softening is observed around 141°C. At about 219°C, the chitosan modulus starts to increase, and this indicates thermal degradation and associated cross-linking reactions. This temperature region is associated with elimination of acetamide and amine groups (Kim et al., 1994). From Figure 8B, chitosan shows major tan delta peaks at 188 and 283°C. The 188°C peak is probably associated with Tg. This seems slightly higher than reported in the study by Ogura et al. (1980), but moisture content or the compositional differences between chitosans may explain the differences. In the composites, chitosan transitions are suppressed by the chitin network.

## Conclusions

Nanostructured chitin-chitosan nanocomposites completely based on crustacean chitin were prepared. In the context of chitin nanocomposites, the present materials showed a unique combination of modulus, strength and strain-to-failure so that the work to fracture (area under stress-strain curve) was as high as 35 MJ/m^3^ at a chitin volume fraction of 8%. Also, at very high chitin content (56 vol%), the nanocomposites showed considerable strength, 140 MPa, and strain to failure, 11%. The high strain-to-failure in the nanocomposites is due to reorientation, slippage and straightening of chitin nanofibers in the ductile chitosan matrix. Combined with the small diameter of the chitin and the favorable chitin-chitosan interface interaction, these factors delay formation of microcracks to very high strain. The favorable interface structure is further supported by the observation that moisture sorption of the composites is lower than for either neat chitosan or neat chitin membranes. Most likely, the original moisture sorption sites at the chitin nanofiber surface are no longer available due to strong molecular chitin-chitosan interactions.

The nanostructured material characteristics were confirmed by microscopy. The nanoscale dimensions of the chitin nanofibers prepared in the present study, as well as the low protein content was confirmed. The largest agglomerates in the materials were in the form of a low fraction of fibrous chitin bundles with a diameter of around 100 nm. The rest of the chitin nanofibers showed a diameter at the scale of 10 nm or less. Colloidal mixtures of chitin nanofibers and dissolved chitosan showed high transparency and good mixing behavior, much better than for the individual components by themselves, and this is essential. Chitin-chitosan repulsion in the colloidal state was confirmed as the main dispersion mechanism. The good colloidal dispersion has favorable effects on chitin nanofiber distribution in the solid material. The nanofibers are well dispersed in the form of curved semi-flexible nanofibers in a chitosan matrix. Fracture surfaces indicate a layered chitin nanofiber structure, and to some extent, flocs are formed as chitin concentration is increased during drying.

Food industry waste in the form of exoskeletons from crab, shrimp, and lobster has potential use in nanostructured chitin/chitosan films of high ductility and strength. In terms of mechanical properties, chitin nanofibers appear to provide better reinforcement effects than chitin nanocrystals due to higher chitin content and the nanofiber network structure. Scientifically, continued focus should be on understanding extraction mechanisms for the nanofibers as well as interface interaction mechanisms in materials containing chitin, chitosan and corresponding counterions. The relevance of published studies on cellulose nanofibers is apparent, and can provide inspiration in future efforts on chitin nanomaterials. Smaller chitin nanofiber diameter, preserved chitin molar mass and tailored chitin-chitosan charge interactions would lead to better chitin dispersion. This is likely to result in high chitin content nanocomposites of even higher toughness than in the present study.

### Conflict of interest statement

The authors declare that the research was conducted in the absence of any commercial or financial relationships that could be construed as a potential conflict of interest.
